# Sodium Channels and Local Anesthetics—Old Friends With New Perspectives

**DOI:** 10.3389/fphar.2022.837088

**Published:** 2022-03-28

**Authors:** Jannis Körner, Simone Albani, Vishal Sudha Bhagavath Eswaran, Anna B. Roehl, Giulia Rossetti, Angelika Lampert

**Affiliations:** ^1^ Institute of Physiology, Aachen, Germany; ^2^ Clinic of Anesthesiology, Medical Faculty, Uniklinik RWTH Aachen, Aachen, Germany; ^3^ Institute for Neuroscience and Medicine (INM-9/IAS-5), Forschungszentrum Jülich, Jülich, Germany; ^4^ Faculty of Mathematics, Computer Science and Natural Sciences, RWTH Aachen, Aachen, Germany; ^5^ Jülich Supercomputing Center (JSC), Forschungszentrum Jülich, Aachen, Germany; ^6^ Department of Neurology, University Hospital Aachen, RWTH Aachen University, Aachen, Germany

**Keywords:** sodium channel, local anesthetics, 3D structural modelling, ion channel, pain

## Abstract

The long history of local anesthetics (LAs) starts out in the late 19th century when the content of coca plant leaves was discovered to alleviate pain. Soon after, cocaine was established and headed off to an infamous career as a substance causing addiction. Today, LAs and related substances—in modified form—are indispensable in our clinical everyday life for pain relief during and after minor and major surgery, and dental practices. In this review, we elucidate on the interaction of modern LAs with their main target, the voltage-gated sodium channel (Navs), in the light of the recently published channel structures. Knowledge of the 3D interaction sites of the drug with the protein will allow to mechanistically substantiate the comprehensive data available on LA gating modification. In the 1970s it was suggested that LAs can enter the channel pore from the lipid phase, which was quite prospective at that time. Today we know from cryo-electron microscopy structures and mutagenesis experiments, that indeed Navs have side fenestrations facing the membrane, which are likely the entrance for LAs to induce tonic block. In this review, we will focus on the effects of LA binding on fast inactivation and use-dependent inhibition in the light of the proposed new allosteric mechanism of fast inactivation. We will elaborate on subtype and species specificity and provide insights into modelling approaches that will help identify the exact molecular binding orientation, access pathways and pharmacokinetics. With this comprehensive overview, we will provide new perspectives in the use of the drug, both clinically and as a tool for basic ion channel research.

## Graphical Abstract

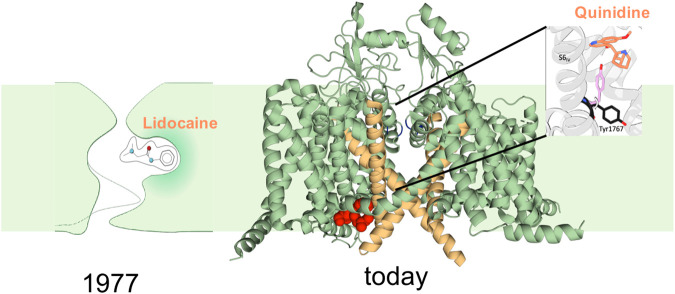

## 1 Introduction

The effectiveness of local anesthetics (LAs) was discovered by the ancient Incas, who used the leaves of the coca-plant to numb pain. This was adapted by Spanish colonialists and the use of cocaine as a LA was documented as early as in 1884 (Freud, 1885). The frequency of toxic reactions to cocaine affecting the central nervous and cardiovascular system grew as fast as the popularity of its use, and the initial euphoria about LAs gave way in time to skepticism. Therefore, the pharmacological industry concentrated on an analysis of natural products in plants in order to find a substitute drug for cocaine that would still be anesthetic but with less side effects. This strategy led to the isolation of tropacocaine in 1891 (Naef, 1994) which unfortunately showed similar levels of toxicity as cocaine. In the next years, variations of cocaine were driving the development of the amino ester LA benzocaine in 1900. With the discovery of procaine in 1905, several other amino amide LAs were produced, including chloroprocaine, cinchocaine, lidocaine, mepivacaine, prilocaine, efocaine, bupivacaine, etidocaine, and articaine. While all these LAs were found to be significantly less toxic than cocaine, they still cause toxic effects in the central nervous system (CNS) as well as cardiovascular toxicity (Yvan et al., 2001). Numerous experimental studies were conducted to try and identify the cellular mechanism of this toxicity, but this in turn required an understanding of the action of LAs. Their mechanism of action, however, would only be identified 60 years later: voltage clamp of the giant squid axon showed that LAs block the fast-gating voltage-dependent sodium current in nerves and muscles (Taylor, 1959).

It is now known that, above all, LAs exert their effects through blockage and gating modulation of voltage-gated sodium channels (Navs). Navs are large membrane proteins, whose α-subunit exists as nine different isoforms (Nav1.1-1.9): Nav1.1, Nav1.2, Nav1.3 and Nav1.6 are expressed in the CNS and peripheral nervous system (PNS); Nav1.7, Nav1.8 and Nav1.9 are predominantly expressed in the PNS; Nav1.4 is expressed in the skeletal muscles while Nav1.5 is the predominant isoform of the cardiac muscles (Körner and Lampert, 2020). In the decades following the identification of Navs as target for LAs, the electrophysiological characterization of Nav modification was performed with great effort and precision (Fozzard et al., 2011).

In recent years the publication of cryogenic electron microscopy (cryo-EM) structures of Navs led to a tremendous progress in our understanding of their structure, function and drug binding relation. Educated assumptions on structural channel features based on valid experimental data could finally be put to test using these new structures.

Besides Navs, other membrane proteins have also been described to be modulated by LAs. Lidocaine activates TPRV1 and to a lesser extent TRPA1 expressed in HEK cells (Leffler et al., 2008). ATP activated potassium channels in dissociated rat myocytes were blocked by bupivacaine with an IC50 of 29 µmol (Olschewski et al., 1999). A study by (Kindler and Yost, 2005) reported that 2-pore domain potassium (K2P) channels are also another target for LAs. Rapidly inactivating potassium currents (RA-currents) in dorsal horn neurons of different laminae from rats could be inhibited by bupivacaine, mepivacaine and lidocaine (Olschewski et al., 1998). Lidocaine, bupivacaine, and tetracaine were shown to block voltage gated potassium currents (transient and sustained) in rat dorsal root ganglia (DRG) neurons (Komai and McDowell, 2001). Blockage of voltage-gated calcium channels in rat sensory neurons was observed when treated with tetracaine (Sugiyama and Muteki, 1994). Lidocaine and QX314 were shown to block the calcium signaling of G-protein-coupled receptors (GPCRs) in *Xenopus laevis* oocytes (Hollmann et al., 2001). NMDA receptors were also shown to be targets of LAs, with bupivacaine blocking these receptors when expressed in HEK cells (Paganelli and Popescu, 2015).

While the targets for LAs range from ion channels to GPCRs, we will focus on Navs in this review, as they are the main site of their action. In the subsequent sections, we will describe the different chemical types of LAs, their mechanism of action on Navs, and, finally, aspects of their clinical use and pharmacokinetics.

## 2 Chemical Structure of LAs

LAs are a family of small organic molecules, with a structure that can be divided in three sections (Becker and Reed, 2012; Shah et al., 2018; Taylor and McLeod, 2020):

I. An Aromatic Group, Usually Containing a Benzene Ring
II. An Amphipathic Ester or Amide Linkage
III. A tertiary amine moiety, acting as base proton acceptor.

This division still reflects the derivation of LAs from cocaine and it shows how this basic scheme was maintained during the many attempts of the last century to create better derivatives (Ruetsch et al., 2001).

**FIGURE 1:**
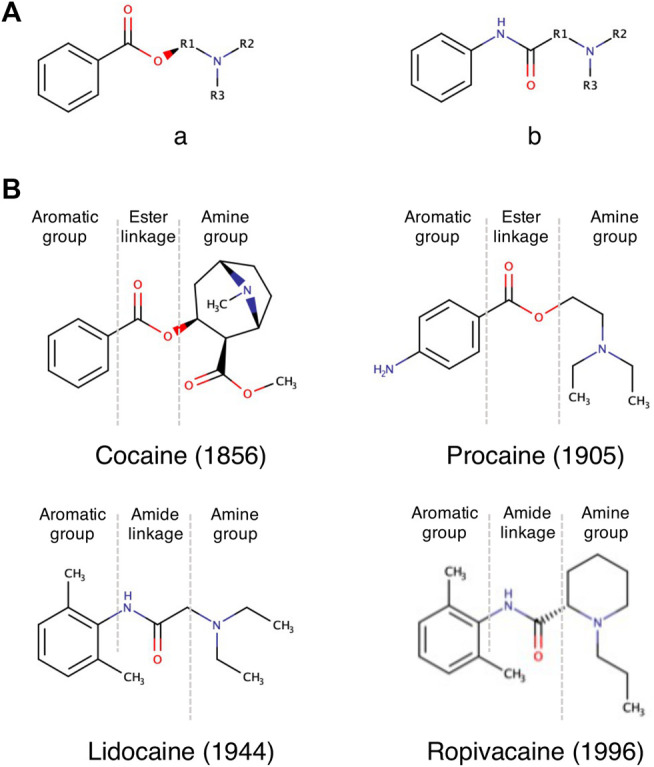
Overview of the chemical structure of LAs. **(A)** LAs consist of two main chemical classes: The lipophilic aromatic ring can be linked to the tertiary amine by an intermediate ester (a) or amide linkage (b). **(B)** Example chemical structures of LAs. The name of the selected LA and their discovery date are given below each structure.

LAs are categorized in two groups based on the chemical nature of the linkage: amino esters (cocaine, tropocaine, eucaine, benzocaine, procaine) and amino amides (lidocaine, mepivacaine, ropivacaine) (Figure 1A). The nature of the linkage determines the way the compound is metabolized by the organism, its chemical stability and its propensity in triggering allergic reactions, with the amide linkage deemed to be the less toxic for the human organism (Shah et al., 2018). Since the development of lidocaine in 1944, all new LAs contain an amide linkage (Ruetsch et al., 2001) (Figure 1B).

The third section of the compound contains an amine moiety that can be protonated at a low pH. This is fundamental in determining the pKa of the LA, which in turn affects the onset and potency of the drug (Becker and Reed, 2012; Shah et al., 2018). Generally speaking, LA molecules, when dissolved in a watery solution, are present in both charged and uncharged states at an equilibrium. As the pKa of most LAs is higher than the physiological pH the charged state is the predominant one. With increasing pH, the fraction of the uncharged state increases as well. This uncharged state has a higher liposolubility and consequently a more rapid diffusion through neuronal membranes, contrasted by a slower diffusion in a patient’s tissue fluids. This leads to a faster onset in *in vitro* experiments with isolated cells or nerve fibers. In a clinical context, this has not necessarily to be the case as high liposolubility can lead to accumulation in neighboring adipose tissues, or the slow diffusion in patients’ tissues can hamper the access to the neuronal membrane (Becker and Reed, 2012) (see chapter 5 below on how this clinically relevant issue can be addressed) Table 1.

**TABLE 1:** Chemical classification of most known LAs. Benzocaine does not have a pKa value as it only exists in the uncharged state.

Name	Class	pKa
Cocaine	ester	8.6
Tropacocaine	ester	9.3
Benzocaine	ester	—
Procaine	ester	8.9
Tetracaine	ester	8.5
2-chloroprocaine	ester	8.7
Dibucaine	ester	8.8
Lidocaine	amide	7.6
Mepivacaine	amide	7.6
Prilocaine	amide	7.9
Bupivacaine	amide	8.1
Etidocaine	amide	7.7
Ropivacaine	amide	8.1
Levobupivacaine	amide	8.1

The difference in distribution with varying pH also needs to be considered when using LAs in inflamed tissue, in which the pH often is lowered. In these conditions, a larger part of the LAs is protonated and does not easily pass into and through the cell membrane to enter the Nav protein and be effective. Thus, in inflamed tissue LAs may not be as effective as expected. The ratio between the number of molecules in the charged and in the uncharged state can also be affected by and the solution in which LAs are stored (Brandis, 2011) or administered (Quinlan et al., 1992; Brandis, 2011), and as well by the temperature the LA is delivered (See chapter 5—clinical perspectives). While the coexistence of differently charged states is true for most LAs, some of these compounds can only exist in an uncharged (e.g., benzocaine, which does not contain an amine tail) or charged (e.g., QX-314) form. This latter group of molecules, containing a permanently protonated quaternary amine, cannot permeate through biological membranes and their efficacy as anesthetics has been debated for years (Strichartz, 1973; Wang et al., 1995; Lim et al., 2007). Nevertheless, recently an elegant approach for its potential clinical use was described as outlined in chapter 5 on the clinical application of LAs.

## 3 The Structure of Navs

**FIGURE 2:**
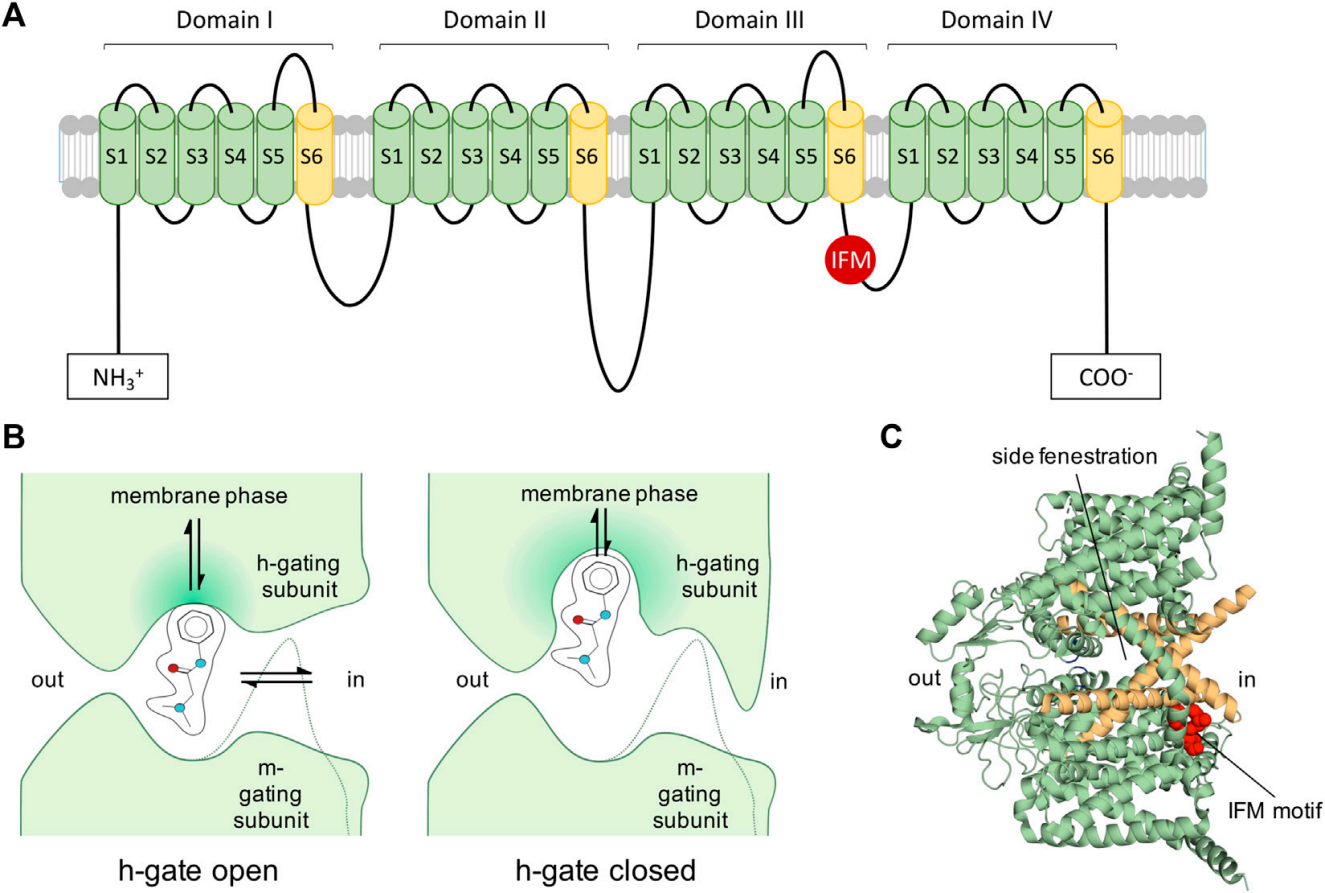
Voltage-gated Navs as targets of LAs. **(A)**: 2D representation of Nav. Inactivation motif is indicated by red circle labelled with IFM (isoleucine, phenylalanine, methionine). **(B)**: View of 1977: Diagram of a LA molecule binding into the pore of a Nav indicating the activation gate (m-gate) and inactivation gate (h-gate) according to an early view and description of Nav modification (Hille, 1977). LAs were assumed to enter *via* the membrane phase or passing the m- and h-gate from the intracellular side. Many assumptions proved to be correct, such as the possibility for LAs to enter into the channel’s pore from the membrane phase. The figure was partially readapted from (Hille, 1977). **(C)**: Cryo-EM structure of hNav1.7 (Shen et al., 2019) (PDB ID: 6J8G) depicting the side fenestrations, which allow LAs to enter the channel from the lipid phase and the inactivation motif (IFM-motif, shown in red), which was previously described as h-gate.

Eukaryotic Navs are complexes of a large, pore-forming α subunit of 250 kDa with one or two β subunits of 30–40 kDa (Hartshorne et al., 1982; Messner and Catterall, 1985); reviewed in (Körner and Lampert, 2020) (see Figure 2). The α subunits are composed of 24 transmembrane segments organized in four homologous domains (I-IV) each containing six transmembrane segments. The six transmembrane segments are mostly α-helical (S1–S6) and form the voltage-sensing (S1–S4) and pore-forming unit (S5–S6), which are connected through the S4–S5 cytoplasmic linker.

When an electrical impulse reaches an excitable cell and depolarizes it enough, Navs open for a few milliseconds and sodium ions can pass (Hodgkin and Huxley, 1952). Within milliseconds after channel opening fast inactivation occurs by binding of an inactivation motif (Vassilev et al., 1988; Ahern et al., 2016). In this state, no sodium ions can permeate. To recover the channel from this refractory state, the cell membrane needs to repolarize, allowing the channel’s voltage-sensors to deactivate and the inactivation particle to dissociate from the channel. From this resting, non-conductive state, the channel can be activated again.

Mutagenesis, electrophysiology, and molecular modeling have contributed to provide a provisional map of the functional parts of Navs: Voltage-dependent activation is initiated by voltage-driven outward movement of the positive gating charges (arginine and lysine residues) in the S4 of the voltage-sensing units, while ion selectivity and sodium conductance is mediated by S5–S6 unit and the connecting P loop (Heinemann et al., 1992; Yang et al., 1996; Payandeh et al., 2011; Clairfeuille et al., 2019; Wisedchaisri et al., 2019; Jiang et al., 2020) reviewed in (Ahern et al., 2016). Within 1–2 ms after opening, the channels undergo fast inactivation. During this process a motif formed by the amino acids isoleucine, phenylalanine, and methionine (IFM) on the intracellular linker connecting domains III and IV interacts with its binding pocket on the pore. This binding most likely happens on the side of the pore and induces an allosteric modulation that closes the permeation pathway, as suggested by several recent cryo-EM structures (Jiang et al., 2021; Shen et al., 2019, 2017) and a follow-up functional mutagenesis investigation (Rühlmann et al., 2020). This allosteric mechanism contrasts the traditional view of a direct block of the pore by the inactivation gate as initially proposed by the hinged-lid or ball-and-chain hypothesis (West et al., 1992; Kellenberger et al., 1996, 1997). During prolonged depolarization, Navs can also enter a slow-inactivated state from which recovery requires prolonged repolarization (Rudy, 1978). Structurally, most likely the pore is responsible for this slow reduction of channel availability (Silva, 2014).

Apart from the transmembrane segments a large portion of the channel protein is located at the extracellular and intracellular regions. The glycosylated extracellular loops are involved in toxin interactions (see chapter 5 below on how this property can be used in a clinical context) and form contacts with the extracellular matrix, while the larger cytoplasmic convolutes are involved in hormonal, cytoskeletal and metabolic regulation (Balse and Eichel, 2018; O’Leary and Chahine, 2018). In contrast, the Nav β subunits are single membrane-spanning glycoproteins with a small intracellular domain and an extracellular immunoglobulin-like domain, similar to cell adhesion molecules (Namadurai et al., 2015). Expression of the α subunit alone is sufficient to reconstitute Nav function in heterologous expression systems, and the β subunits modify the kinetics and voltage dependence of Nav activation and inactivation as well as trafficking (Isom, 2001).

The three-dimensional structure of the core functional unit of a voltage-gated Nav was firstly solved for the ancestral homotetrameric bacterial Nav of *Arcobacter butzleri* (NavAb) by X-ray crystallographic studies in a pre-open (PDB ID: 3RVY) (Payandeh et al., 2011) and slow-inactivated state (PDB ID: 4EKW) (Payandeh et al., 2012). Bacterial Nav channels share important key features with their eukaryotic descendants, but their simpler homo-tetrameric structures lack a fast-inactivation gate. Therefore, they undergo an inactivation process similar to slow rather than fast inactivation (Pavlov et al., 2005). When examining the NavAb structure from the membrane side, it also revealed the existence of additional lateral lipid-facing openings, called fenestrations, connecting the exterior of the channel protein with the central pore. These channel fenestrations could create pathways for small hydrophobic blockers to access the pore from the side through the membrane. It was indeed observed that in both cryo-EM structures in the closed and potentially slow inactivated state, the lateral fenestrations of the channels are open, possibly enabling the entrance of hydrophobic molecules to the central cavity of the channel (O’Reilly et al., 2012; Payandeh et al., 2012, 2011).

Soon after the publication of the bacterial Nav structure, cryo-EM analysis of eukaryotic nerve and skeletal muscle Nav complexes provided significant insights into the overall structure of eukaryotic Navs. The channel from the American cockroach *Periplaneta americana* (PDB ID: 5X0M) (NavPaS) was the first eukaryotic Nav to be observed at near-atomic resolution (Shen et al., 2017). It comprises of a single-chain pseudo-tetramer and proves to have a sequence very similar to most vertebrate Nav channels. While the IFM motif seems to be replaced by the aminoacids ATD, the inactivation process may still occur in a similar fashion as in mammalian Navs (Shen et al., 2017). The structure of the functional core is very similar to NavAb. The backbones of the pore and ion selectivity filter (SF) in the center of these structures are essentially identical to their bacterial ancestors. An important difference is at the level of the SF: while selectivity in NavAb is entailed by four glutamate residues (EEEE), the SF of NavPas is made from the aminoacids DEKA (Heinemann et al., 1992). Also in the NavPaS structure a possible access path for hydrophobic molecules at the membrane side of the channel was identified, but this time, because of the intrinsic asymmetry of the channel, only one open fenestration between domain III and IV can be seen (Shen et al., 2017). NavPaS was snapped in a closed-pore state, with the VSDs in an activated, “up” position.

In 2017, the structure of Nav1.4 from *Electrophorus electricus* (Electric eel, eeNav1.4), a functionally well-established channel was solved at 4.0 Å resolution (PDB ID: 5XSY) (Yan et al., 2017). The structure has all the four fenestrations in an accessible state and its fast-inactivation is mediated by an LFM motif in the DIII-IV linker (Yan et al., 2017), homologue to the IFM motif observed in human channels. In the eeNav1.4 structure, the LFM is plugged in the corner between DIII and IV in contact with S4-5 and S6 of both repeats, suggesting that the inactivation occurs via an allosteric blocking mechanism (Yan et al., 2017).

Binding of the inactivation motif was observed in more detail in the human Nav cryo-EM structures published since 2018, namely the structures of subtypes hNav1.4 (PDB ID: 6AGF) (Pan et al., 2018), hNav1.5-E1784K (PDB ID: 7DTC) (Li et al., 2021b) and hNav1.1 (PDB ID: 7DTD) (Pan et al., 2021), which show the IFM motif plugged into a hydrophobic cavity formed by the segments S4-S5 and S6 from repeats III and IV and S5 from repeat IV. The allosteric effect of the inactivation motif was compared to a door wedge. By entering the cavity, the motif pushes against the S6 and the intracellular gate restricts (Li et al., 2021b). Evidence from mutagenesis studies supports this new function of fast inactivation (Rühlmann et al., 2020). A more recent cryo-EM structure of the rat Nav1.5 in a putative open state with the IFM motif mutated to three glutamines (rNav1.5 IFM-QQQ), provides further proof for the allosteric mechanism of fast inactivation: in this mutant the DIII S6 and DIV S6 to move away from the pore axis, with DIV S6 moving towards the hydrophobic cavity where the IFM motif would bind (Jiang et al., 2021).

The lateral side fenestrations in Navs were speculated to play a role in hydrophobic access of molecules such as the LAs to the pore, provided credence by open lateral fenestrations in the early NavAb structures (Payandeh et al., 2012, 2011). With the increasing number of crystal structures of eukaryotic Navs being published over the recent years, it has become possible to study the differences in size and conformation of the side fenestrations in these various cryo-EM structures. The results are not entirely straight-forward and we summarize the key findings from such studies below. Comparison of the NavPaS structure with the mammalian voltage-gated calcium channel Cav1.1 (PDB ID: 5GJV) revealed that the NavPaS VSDs of DI, DIII and DIV were in a relatively more ‘downward’ conformation than Cav1.1 (Shen et al., 2017). In contrast to NavPaS (which has only one narrow opening at the DIII-DIV interface), all side fenestrations of Cav1.1 were seen to be open (Shen et al., 2017). The same was true in the case of the eeNav1.4 and hNav1.4, showcasing four wide open fenestrations (Yan et al., 2017; Pan et al., 2018). The hNav1.7 shows a closure of only the DI-DIV fenestration, due to a different rotamer of M1754 compared to hNav1.4 (Shen et al., 2019). Modification of a crucial phenylalanine residue at the entry of the fenestration in NavAb either to a bulkier tryptophan (PDB ID: 6MVW) or a smaller alanine (PDB ID: 6MVV), can decrease or increase the size of the fenestrations respectively (El-Din et al., 2018). The relevance of aromatic residues in determining the size of side fenestration was recently supported by data from human Navs as well by using molecular dynamics (MD) simulations (Tao and Corry, 2022). In their study, the authors applied MD simulations on different Navs subtypes and discover that the fenestration size of DI-DII and DIII-DIV seems to be wider than the DII-DIII fenestration, with a more restricted DI-DIV fenstration (Tao and Corry, 2022). A limitation of the study (caused by a lack of hNav structures in different gating states) was the usage of hNav structures only in the inactivated state. There is evidence at least in prokaryotic channels that the size of the fenestrations can change with the functional state of the channel (Catterall et al., 2020; Payandeh et al., 2012, 2011). It is not far-fetched to consider the possibility that also in the case of the eukaryotic Navs, the size and shape of the fenestrations depend on the state of the channel and also vary between the different domain interfaces delimiting the fenestrations (e.g., DIV-DI vs. DI-DII). As LAs may use the side fenestrations as entry pathway (see below), this may be relevant for their state dependent activity.

Navs have also been captured with bound compounds and toxins, i.e. the cryo-EM hNav1.7 structures in combination with tetrodotoxin and protoxin-II or with saxitoxin and huwentoxin-IV (PDB IDS: 6J8G, 6J8H, 6J8I and 6J8J) (Shen et al., 2019). In some of these cases, the compounds belonged to the LA family, as in the case of the cryo-EM structures of hNav1.5 + Quinidine (PDB ID: 6LQA) (Li et al., 2021b), rat Nav1.5F + Flecainide (PDB ID: 6UZ0) (Jiang et al., 2020) , rat Nav1.5 + propafenone (PDB ID: 7FBS) (Jiang et al., 2021) or the Xray structures of NavAb + lidocaine (PDB ID: 6MVY) and NavAb + flecainide (PDB ID: 6MVX) (El-Din et al., 2018). The LA binding sites will be discussed in detail using these last group of structures in Section 5.

## 4 Mechanisms Underlying LA Channel Block and Gating Modifications

LAs inhibit electrical nerve impulses by selectively interfering with the function of Navs and thus blocking the sodium ion permeation over the cell membrane. As this is needed for the fast upstroke of the action potential, LAs effectively inhibit impulse propagation and thus nociceptive (and other) signaling.

The interplay between the LAs and the channel protein is complex and a simplified view of the drugs blocking the pore like a cork in a bottle would not live up to its intricacies. Profound research has been performed on the blocking mechanisms of LAs with both functional and structural approaches. Thereby a partly variable nomenclature for the different blocking mechanisms of LAs was used and we aim to clarify this in the following paragraph.

Already in 1973 Stricharts (Strichartz, 1973) performed experiments involving repetitive stimulation of Navs exposed to the polar LA substance QX-314. In 1975 Courtney summarized these findings very accurately by explaining “…that the influence of this drug is remarkably dependent upon the previous history of use of Navs.” (Courtney, 1975), meaning that repetitive stimulation (use) of Navs increased the LA-mediated (in this case QX-314) decrease in sodium conductance (Strichartz, 1973). Consequently, this block was termed “use dependent” or “phasic” block. In contrast, the less effective LA-mediated block occurring from the channel’s resting state, i.e., at hyperpolarized potentials (usually −120 mV) and without repeated prior channel activation, is referred to as “resting” or “tonic” block. The use dependent characteristic seems ideal to treat states of hyper-excitability—only in the pathological state of increased neuronal activity, the drug would block the channels and thereby silence e.g., the activated nociceptor, while a low frequency activity would reduce the channel’s affinity to the LAs.

This reliance of the LA binding on Nav gating cycles gained another dimension when it became clear that the block was also dependent on the chemical structure and the site of application of the blocker. Use dependent block was most pronounced for quaternary substances, when applied intracellularly, leading to the concept that the LA binding site was only accessible from the cytoplasmatic side and when the channel was in the open state (Strichartz, 1973). Several studies focused on these phenomena. For instance, QX-314, a permanently charged LA, quickly blocked the channel when repetitively activated only when placed to the intracellular—but not the extracellular—side. Observations performed by Courtney on the behavior of GEA 968, a hydrophilic LA, produced similar results, showing that depolarization was necessary to block the channel (Courtney, 1975). Taken together, this suggested that charged or hydrophilic molecules are only able to bind to the open state of the channel.

Another dimension to be included in the understanding of sodium channel local anesthetic interaction was that the block appears to be interfering with the fast inactivation process itself. Early studies describe a much prolonged recovery from inactivated states upon treatment with procaine (Khodorov et al., 1975, 1974) or GEA 968 (Courtney, 1975).When stimulated while LAs are present, channels were shown to accumulate in the inactivated gating state (Courtney, 1975) resulting in a shift of the voltage dependency of inactivation to hyperpolarized potentials. On this basis Courtney suggested a model for the interaction of Navs and LAs in which the transition between blocked and unblocked states can happen the most readily when the channel is in the open state (Courtney, 1975). As the inactivated state of blocked channels is more stable and recovery is hampered compared to the inactivated state of unblocked channels, repetitive stimulation leads to cumulative block, called the use-dependent block (Courtney, 1975).

**FIGURE 3:**
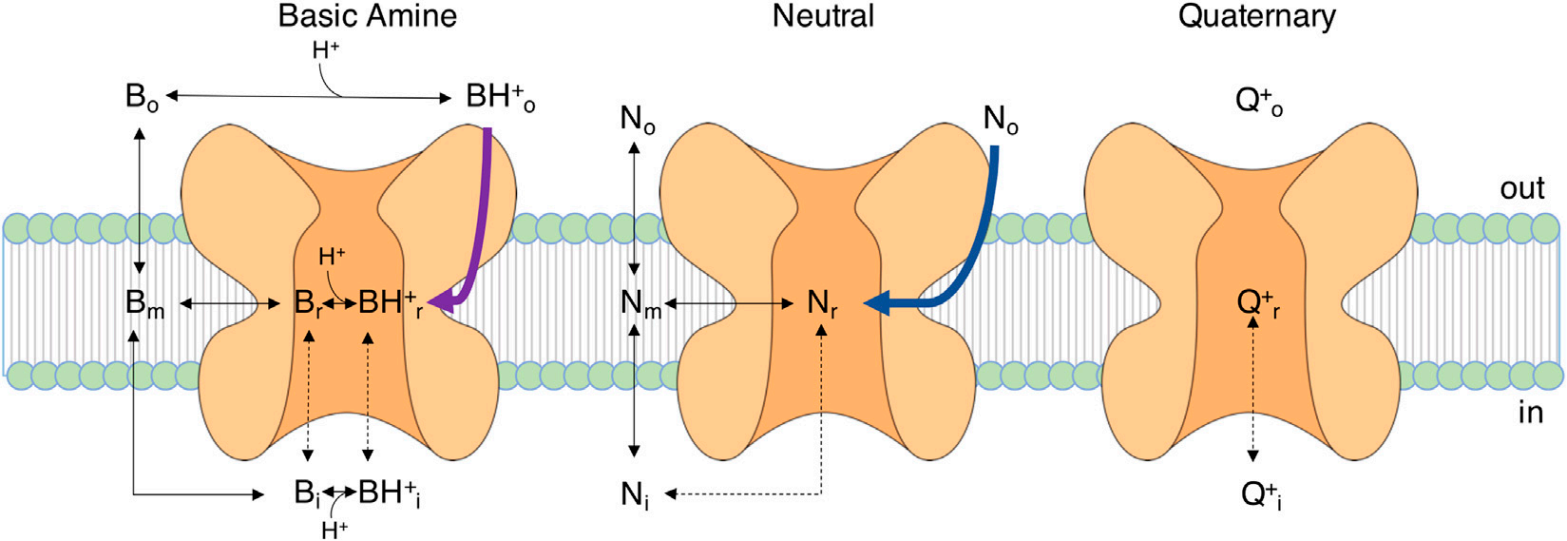
Entry pathways of LAs with different chemical properties. Diagram of the movements of differently charged LA molecules (B, basic amine, N, neutral LA, Q, quaternary charged LA) to and from the channel. Black dashed lines are transitions requiring open activation and fast inactivation gates, as the drug enters from the intracellular side. Black solid lines are transitions probably not strongly depending on the gating state. Violet and blue lines indicate two pathways observed in MD studies for lidocaine and benzocaine, respectively, (Boiteux et al., 2014; Nguyen et al., 2019), where the LA does not pass through the membrane phase but enters a lipophilic pathway within the channel protein towards the side fenestrations (see also Figure 4). Subscript characters represent membrane phase (m), receptor site (r), extracellular (o) and intracellular (i) space. The figure is based on the diagram reported in (Hille, 1977).

In 1977, Hille further investigated the impact of charge of LAs on their blocking behavior. He made the way paving observation that lidocaine, tetracaine or benzocaine (which are basic amines) induced a hyperpolarizing shift of steady-state fast inactivation also without repetitive stimulation, suggesting that these amphiphilic LAs find access to the channel also in the closed state. The charged, quaternary analog GEA968, on the other hand, could only exert its effect on Navs when trains of pulses were applied, suggesting that channel access for charged LAs needs the activation gate to open. (Courtney, 1975; Hille, 1977). In other words, transitions between unblocked and blocked resting (R->R*) or inactivated (I->I*) channels (“normal states” and “modified states” in his model) were restricted to hydrophobic and amphiphilic drugs. Transitions between unblocked and blocked open channel states (O->O*), on the other hand, are possible both for hydrophilic and hydrophobic compounds (see also Figure 3). We will discuss below how these differences in access are defined structurally.

### 4.1 Modulated Receptor Hypothesis

All these studies on LA effects on Navs were condensed in the seminal work of Hille in 1977 in which the mechanism of action of LAs was explained with the modulated receptor hypothesis (Hille, 1977), based on earlier works describing similar phenomena in potassium channel drug interactions (Armstrong, 1966). Within this concept there is only one LA receptor site, and its affinity or accessibility is dependent on the gating state of the channel. Substances show distinct affinity to specific gating states resulting in varying fractions of resting and use dependent blocks. Hille claims the existence of at least two access pathways to the LA receptor site:

I. The hydrophilic pathway from the intracellular side of the channel which is only accessible when the channel’s activation gate is open. This access pathway is strongly voltage-dependent leading to pronounced use dependent characteristics of block.
II. The lipophilic pathway, where the drug enters the channel from its side facing the lipid phase of the membrane. This access route is not dependent on the state of the activation gate and thereby less use dependent (Hille, 1977).

Consequently, the use-dependent, phasic block (I) is also called hydrophilic while the resting, tonic block (II) is also called lipophilic. The modulated receptor hypothesis assumes altered inactivation kinetics in drug bound channels, thus the mathematical description of the model is relatively complex, as it needs several sets of rate constants for the transitions of the various bound and unbound gating states to describe its function.

### 4.2 Guarded Receptor Hypothesis

In 1984, an alternative to the modulated receptor hypothesis was suggested, called the guarded receptor hypothesis (Starmer et al., 1984). In contrast to the modulated receptor hypothesis, where affinity of the LA binding site is assumed to be a function of the channel state, the guarded receptor hypothesis postulated that the accessibility of the binding site for the LAs is dependent on the channel state but the affinity of the receptor itself remains unaltered. With this concept the gating transitions of the blocked and unblocked states were also mathematically more easily described [Here, only one set of rate constant was estimated and sufficient to simulate use dependent block (Starmer et al., 1986), whereas the modulated receptor hypothesis depends on altered inactivation kinetics in drug bound channels]. The model is working with less parameters than the modulated receptor hypothesis and is able to predict many Nav-blocker interactions correctly. This included the shifts in the voltage dependency of fast inactivation without the need of modification of inactivation gate kinetics by LAs. Nevertheless it has been shown that substances indeed do alter gating inactivation kinetics of Navs (Hanck et al., 1994). Drugs may also bind to the open channel state and then get trapped in the pore by the closing fast inactivation gate. This may thus induce a high-affinity binding with use dependent properties, thus altering the voltage-dependencies of fast inactivation.

### 4.3 LAs Modify Nav Gating

Beside occluding the pore via steric occlusion leading to decreased sodium conductivity several studies investigated by which mechanisms LAs are modifying gating of Navs. It was suggested that LAs would stabilize Navs in an inactivated state: the S4 of DIII and partly DIV, which is involved in fast inactivation as recently also shown by a profound structural study (Clairfeuille et al., 2019), were stabilized in the outward position by LA binding, which was not the case for DI and DII (Sheets and Hanck, 2003). This could lead to a lowered energy barrier for fast inactivation offering a possible explanation why LAs produce hyperpolarizing shifts—which means an enhancement—of the voltage dependency of fast inactivation. One uncertainty of these data is that the effect was more pronounced on DIII whereas the movement of DIV is crucial for fast inactivation. On the other hand, experiments have shown that mutations in DIII, which disrupt the outward stabilization by lidocaine do not alter use dependent block characteristics (Arcisio-Miranda et al., 2010).

Not only mutagenesis but also enzymatic cleavage of the IFM motif resulted in loss of phasic use-dependent inhibition (Cahalan, 1978; Wang et al., 1987). Nevertheless, the assumption that Nav fast inactivation is necessary for frequency dependent LA block did not remain unchallenged as summarized in (Nau and Wang, 2004): 1) chloramine-T, which impairs fast inactivation not by cleaving off the inactivation particle, did not reduce use-dependency (Wang et al., 1987). 2) mutants lacking fast inactivation have been shown to be blocked by mexiletine and flecainide phasically (G. K. Wang et al., 2004; Wang and Wang, 2003). Using a conformational marker for fast inactivation in the rat skeletal muscle Nav it was shown that lidocaine did not compete with the closure of the fast inactivation gate and, strikingly, that recovery from fast inactivation antecedes recovery to ion conductivity in lidocaine bound channels (Vedantham and Cannon, 1999). In addition, it was suggested that lidocaine induced a slow inactivated state (Chen et al., 2000).

It should be noted that much of the data collected to study the interplay between LAs and fast inactivation focused on the inactivation motif in the light of the concept that this motif was sterically occluding the pore (as h-gate or inactivating gate) before the recent insights in cryo-EM studies led the above-mentioned allosteric model of fast inactivation. Isolation of different functional modules of Nav and LA interaction is challenging as channel conformation is voltage dependent and overlaps in dissociation times and channel conformation changes can interfere with the proper analysis of state-, voltage- and substance dependent pharmacodynamics. Nevertheless, it appears evident that the mechanism of action of LAs involves both steric occlusion of the pore and gating modification of the channel.

### 4.4 Open Channel Block or Flickering Block

When Navs are pharmacologically transferred in a non-inactivating state (e.g., by the use of the frog-derived toxin batrachotoxin) LAs and antiarrhythmic drugs may induce a rapid “flickering” between conductive and non-conductive states (Kohlhardt et al., 1989; Zamponi et al., 1993). This open channel block is also called “flicker-block” in these studies and has been shown to be of low affinity when elicited with batrachotoxin (Kd around 4 mM). In experiments on non-inactivating channels, however, the results are not congruent: When investigating open channel block by lidocaine with a IFM/QQQ mutant the IC50 of open channel block is reported to be 400 µM (Bennett et al., 1995). At the same time, when the inactivation deficient mutant rNav1.4-L435W/L437C/A438W (WCW mutant) was investigated, the IC50 is reported to be significantly lower, namely 21 µM (S.-Y. Wang et al., 2004). While the difference between mutagenesis and pharmacological results can be explained with a potentially overlapping binding site between lidocaine and batrachotoxin (Finol-Urdaneta et al., 2018) the differences between the mutagenesis studies remain not fully understood. Navs with intact fast inactivation only open for milliseconds, thus the physiological significance of open channel block by LAs in WT Navs is not trivial to assess.

### 4.5 LA Access Pathways to Their Binding Sites

With the advent of the structural investigations due to the new availability of reliable Nav structures, the far-sightedness of Hille’s LA binding and Nav gating model became apparent: Some of the access pathways predicted by his model could directly be observed during all-atoms MD simulations by using the now published structures. For example, the LA mexiletine was observed entering a homology model of hNav1.5, based on the NavAb structure, *via* the fenestration, when subjected to a pulling force towards the pore in a form of biased simulations called steered MD (O’Reilly et al., 2012).

**FIGURE 4:**
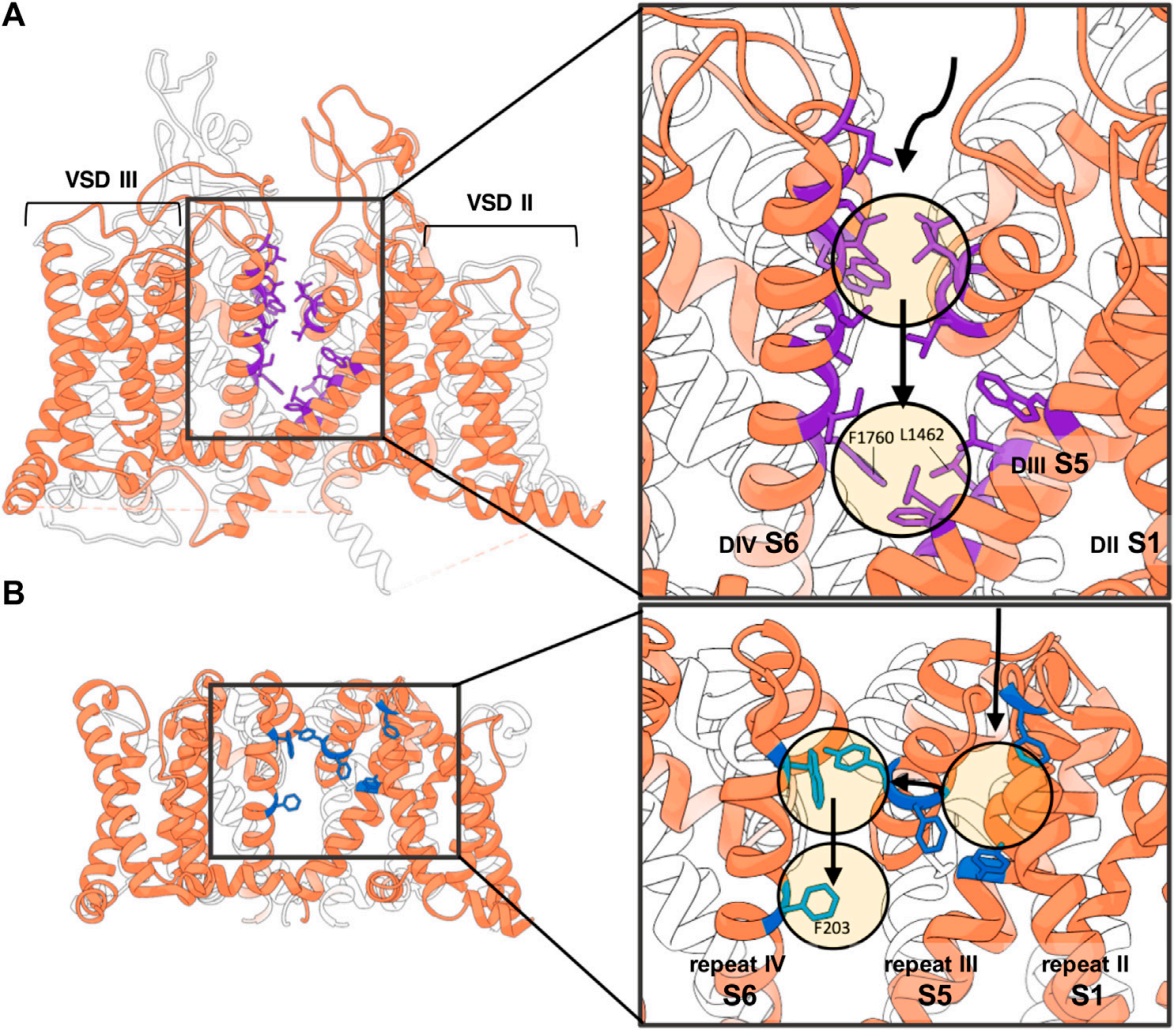
Non-membranous lipophilic LA access via side fenestrations. **(A)** In the hydrophobic access pathway to the pore of the hNav1.5 homology model according to the simulations of (Nguyen et al., 2019) the LA molecule is stabilized along its trajectory by the following residues, colored in purple: L1338, L1342, and W1345 (in DIIIS5); L1410, L1413, and Q1414 (in P1 helix of DIII); L1462 and F1465 (in DIIIS6); W1713, L1717, and L1721 (in P2 helix of DIV); and I1749, T1753, I1756, and I1757 (in DIVS6); and at the entrance of the fenestration F1760 (in DIVS6) and L1462 (in DIIIS6) (Nguyen et al., 2019). Numberings are based on hNav1.5 sequence. **(B)** Lipohilic pathway of benzocaine accessing the pore domain of NavAb *via* the side fenestration. Along its trajectory it encounters low-energy hydrophobic pockets formed by the following residues colored in blue: F37, F142, F167, Y168, W195 and F203 (Boiteux et al., 2014). Numberings are based on NavAb sequence.

In 2014, the NavAb channel from *Arcobacter butzleri* was simulated in the presence of benzocaine, a neutral LA. Benzocaine was observed entering the pore *via* both the intracellular gate and the side fenestrations with the channel gate being closed (Figure 3) (Boiteux et al., 2014). Notably, even though LAs can diffuse from the aqueous phase into the lipid membrane (Bernardi et al., 2009; Boiteux et al., 2014; Mojumdar and Lyubartsev, 2010; Nguyen et al., 2019), in this modelling study they enter the fenestration site from the aqueous extracellular space via an alternative pathway (Figure 4). The authors underlined that this access pathway is stabilized by the presence of transient binding sites along a corridor mostly characterized by hydrophobic residues (Boiteux et al., 2014). A more in-depth study of the same year simulated the entrance of benzocaine *via* NavAb side fenestration and assessed the energetics of ligand binding using three different free energy estimation techniques (Martin and Corry 2014). The study found that benzocaine can travel across the fenestrations on a pathway characterized by low energy barriers. The molecule moves from its preferred position near the lipid headgroups, sliding towards the mouth of the fenestration while maintaining contact with the protein surface (Martin and Corry, 2014).

More recently, a similar pathway from the aqueous extracellular space to the pore was observed when simulating a homology model of hNav1.5 based on eeNav1.4 in the presence of charged and uncharged lidocaine (Nguyen et al., 2019). For positively charged lidocaine molecules, no entrance in the channel cavity was observed. Neutral lidocaine instead was observed to surprisingly enter the cavity via the intracellular gate of the inactivated channel, besides the DIII-DIV fenestration site (Nguyen et al., 2019). Similar to Boiteux et al., in this simulation study the LA molecule accesses the channel side from the extracellular aqueous space and not from the lipid membrane phase, following a pathway comparable to the one described above for benzocaine. This pathway in hNav1.5 is stabilized by transient binding to hydrophobic residues on the P1 helix of DIII (L1410, L1413, Q1414), on the P2 helix of DIV (W1713, L1717, L1721), on DIII S5 (L1338, L1342, W1345), on DIII S6 (L1462, F1465) and on DIV S6 (I1749, T1753, I1756, I1757, F1760) (all hNav1.5 numbering). Notably, only access through the DIII-DIV fenestration site was observed suggesting a preferential pathway for this fenestration. The authors suggest that the neutral lidocaine might favor such pathway due to the absence of bulky residues at the entrance of the DIII-DIV fenestration site as opposed to the other access pathways (Nguyen et al., 2019). It should be noted, that with these kinds of techniques, although useful in identifying possible entrance pathways, do not sample the complete set of possibilities or only do so when the produced trajectory is long enough. The prediction of how probable each entrance pathway is will also be affected by the length of the simulation.

The pathways observed during these simulations suggest that several types of lipophilic entrance ways to the binding site *via* the side fenestration exist: either the molecules enter sideways, from the membranous side [as observed in MD studies of general anesthetics, sevoflurane, (Barber et al., 2014) or isoflurane, (Raju et al., 2013) or mexiletine (O’Reilly et al., 2012)] or they are moving through the channel protein from the extracellular aqueous space as seen for lidocaine in (Boiteux et al., 2014; Nguyen et al., 2019).

Modification of this non-membranous lipophilic pathway between the aqueous extracellular space and the fenestration site by introducing smaller and more polar residues enabled access also to charged blockers in experimental studies: Reducing lipophilicity in the rat muscle Nav1.4 (rNav1.4), by substituting the residue C1572 in this pathway with threonine and I1575 with alanine allowed block by the charged QX-222 molecule (Sunami et al., 2000). Similarly, in rNav1.5 substituting the polar residue T1755 with valine had the same effect, allowing the charged QX-314 to block the channel (Qu et al., 1995). Interestingly, the residue T1755 in rNav1.5 is a valine in both hNav1.2 (V1757) and hNav1.7 (V1730), suggesting that the non-membranous lipophilic pathway may be more accessible for charged molecules in these two channels. Thus, the residues along this non-membranous lipophilic pathway show subtype specificity and may thus provide an explanation for the observed Nav1.5 specific differences in blocking and recovery (Lee et al., 2001).

## 5 LA Binding Sites

The LA binding site has been object of intense research and could be pinned down to a remarkable extent. As the numbering between Nav subtypes differs, we summarize in Table 2 the residues of the different Nav subtypes important for LA binding mentioned in this section to the hNav1.7. We are using isoform 3 of hNav1.7, which is a splice variant in exon 11 that has a deletion of 11 amino acids in the DI-DII intracellular linker (Raymond et al., 2004; Chatelier et al., 2008). This isoform of hNav1.7 was first cloned from the human medullary thyroid carcinoma cell line, and its transcripts were found to be present only in neuroendocrine cells (Klugbauer et al., 1995). With Nav1.7 being a primary target for LAs and isoform 3 widely used in many functional studies of hNav1.7 (Xiao et al., 2010; Theile et al., 2011; Walker et al., 2012; Wang et al., 2015; Körner et al., 2018; Rühlmann et al., 2020) the hNav1.7 isoform 3 is chosen as the reference numbering for Table 2.

**TABLE 2:** Mapping of the residues involved in LA binding of different eukaryotic Nav subtypes (isoforms 1) discussed in Section 5 to the human Nav1.7 (isoform 3). Multiple sequence alignment was performed using the T-Coffee package provided by JABAWS (Troshin et al., 2011). A red color is used to highlight the residues mentioned in Section 5. r represents rats and h humans. The mapping of the residues to the respective regions in the protein are based on the topology of hNav1.7 given in Uniprot (Uniprot ID: Q15858). DI-DIV represent the four domains, S1—S6 represent the six transmembrane segments for each domain, and PM represents the pore module that forms the SF.

Region	hNav1.7	rNav1.2	rNav1.3	rNav1.4	rNav1.5	hNav1.4	hNav1.5
DI-PM	T359	T382	T381	T398	T371	T404	T370
DI-PM	Q360	Q383	Q382	Q399	Q372	Q405	Q371
DI-S6	I386	I409	I408	I425	I398	I431	I397
DII-PM	C914	C940	C892	C753	C899	C759	C896
DII-PM	G915	G941	G893	G754	G900	G760	G897
DIII-S5	L1314	L1341	L1290	L1157	L1340	L1164	L1338
DIII-S5	L1318	L1345	L1294	L1161	L1344	L1168	L1342
DIII-S5	W1321	W1348	W1297	W1164	W1347	W1171	W1345
DIII-PM	L1386	L1413	L1359	L1228	L1412	L1235	L1410
DIII-PM	L1389	L1416	L1362	L1231	L1415	L1238	L1413
DIII-PM	Q1390	Q1417	Q1363	Q1232	Q1416	Q1239	Q1414
DIII-S6	L1438	L1465	L1411	L1280	L1464	L1287	L1462
DIII-S6	F1441	F1468	F1414	F1283	F1467	F1290	F1465
DIII-S6	I1442	I1469	I1415	I1284	I1468	I1291	I1466
DIV-PM	W1689	W1716	W1662	W1531	W1715	W1538	W1713
DIV-PM	L1693	L1720	L1666	L1535	L1719	L1542	L1717
DIV-PM	L1697	L1724	L1670	L1539	L1723	L1546	L1721
DIV-S6	I1726	I1753	I1699	I1568	I1751	I1575	I1749
DIV-S6	V1730	V1757	V1703	C1572	T1755	C1579	T1753
DIV-S6	Y1732	Y1759	Y1705	Y1574	Y1757	Y1581	Y1755
DIV-S6	I1733	I1760	I1706	I1575	I1758	I1582	I1756
DIV-S6	I1734	I1761	I1707	I1576	I1759	I1583	I1757
DIV-S6	F1737	F1764	F1710	F1579	F1762	F1586	F1760
DIV-S6	V1739	V1766	V1712	I1581	I1764	I1588	I1762
DIV-S6	V1740	V1767	V1768	V1582	V1765	V1589	V1763
DIV-S6	N1742	N1769	N1770	N1584	N1767	N1591	N1765
DIV-S6	Y1744	Y1771	Y1772	Y1586	Y1769	Y1593	Y1767

In 1994 Ragsdale and colleagues were the first to functionally characterize the LA-binding site. They performed extensive site directed mutagenesis studies in rat brain Nav1.2 (rNav1.2) expressed in *Xenopus laevis* oocytes and assessed the effect of etidocaine (a tertiary amine LA) on tonic and phasic block (Ragsdale et al., 1994). Alanine substitutions of five amino acids within the channel pore of rNav1.2 were identified to alter tonic block, of which four (I1761, V1766, V1767, N1769) rendered the channel more sensitive to tonic block and one (F1764) reduced it. Distinct (Y1759, I1760) and overlapping (I1761, F1764, V1766, V1767) amino acid substitutions within the pore were identified to affect phasic block. Most of them (Y1759, I1760, I1761, F1764, V1766, V1767, Y1771) resulted in a reduced block when stimulated with 20 pulses at 2 Hz. Of these, I1760, F1764 and Y1771 stand out, as they affected the block most strongly (Ragsdale et al., 1994).

Follow up studies involving DI and DII showed an alanine substitution of a residue in DIS6 of rNav1.2 (I409) to decrease the inactivated state block by etidocaine sixfold (Yarov-Yarovoy et al., 2002) and residues in DIIIS6 of rNav1.2 (L1465, I1469) to be involved in LA-binding (Yarov-Yarovoy et al., 2002). Taken together the data suggest different binding sites for low and high affinity Nav block. Concordantly, for hNav1.5, replacement of F1760 strongly reduces the high affinity channel block (Hanck et al., 2009). The high-affinity binding sites for use dependent block, especially the highly conserved phenylalanine in DIVS6, have been characterized in more detail. Mutagenesis studies in rat Nav1.3 (rNav1.3) indicated that for block of the open or activated state an aromatic residue at this corresponding phenylalanine position F1710 was necessary (Li et al., 1999). By incorporation of unnatural phenylalanine derivates with reduced negative electrostatic potential it has been shown that high affinity use-dependent block of rNav1.4 is determined by cation-π interactions with the highly conserved phenylalanine residue F1579 but interestingly not with Y1586 (Ahern et al., 2008).

**FIGURE 5:**
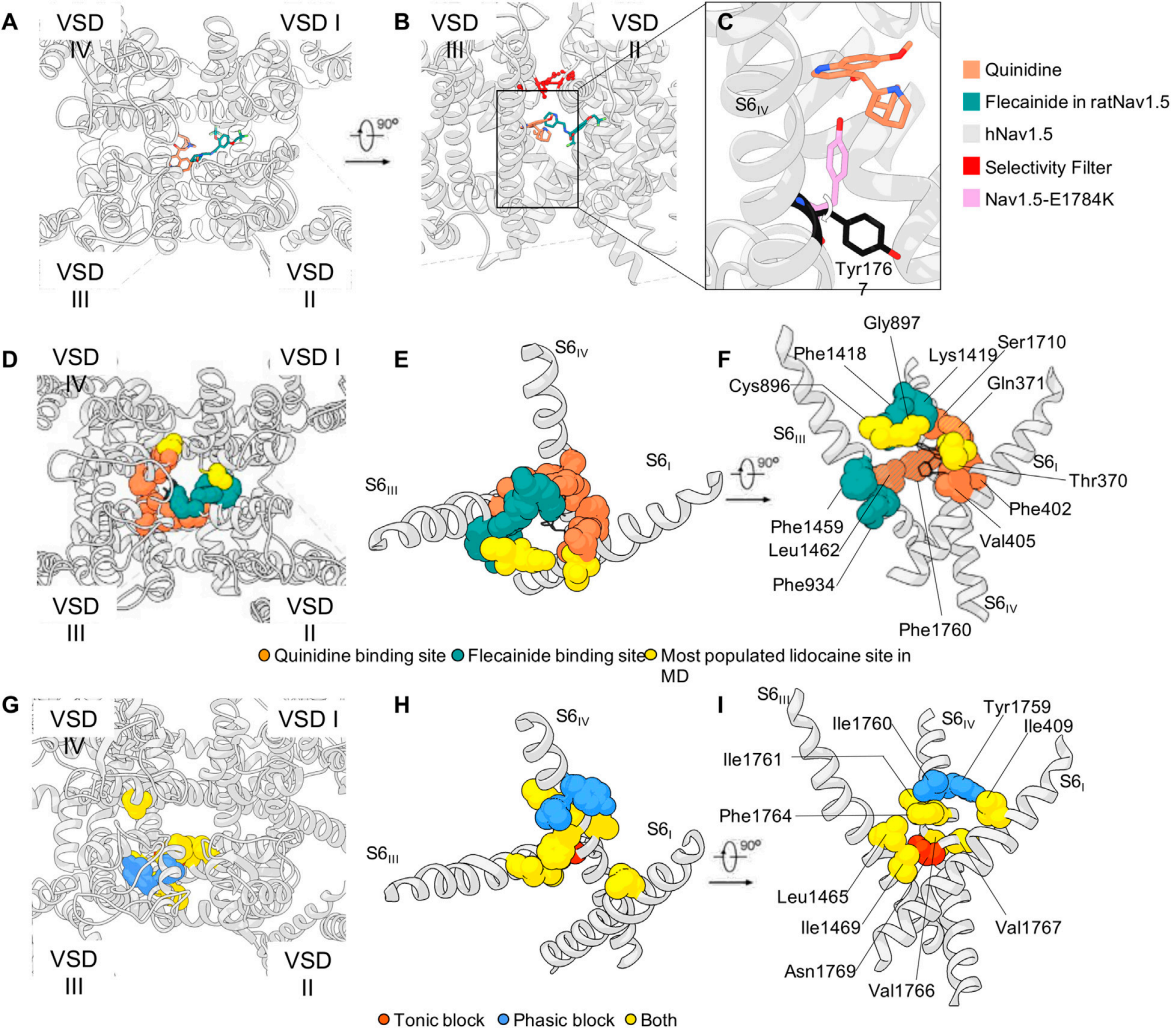
LAs binding sites. **(A,B)** Quinidine in hNav1.5 and flecainide binding poses in rNav1.5 (PDB IDs: 6LQA and 6UZ0, respectively) and **(C)** quinidine’s effect on the rotation Tyr1767, as observed in hNav1.5 (PDB ID: 7DTC). **(D–F)** Sphere representation of the residues that appear in the binding site of quinidine (black wires) [in PDB ID: 6LQA (Li et al., 2021a)], and the corresponding hNav1.5 residues that were additionally observed in the binding pose of flecainide [PDB ID: 6UZ0 (Jiang et al., 2020)], and the lidocaine molecules simulated in (Nguyen et al., 2019). The overlapping residues are colored in stripes in Panel F. **(G–I)** Rat Nav1.2 homology model (https://alphafold.ebi.ac.uk/entry/P04775) and sphere representation of the residues characterized in mutagenesis experiments (Ragsdale et al., 1994; Yarov-Yarovoy et al., 2002). Panels D-F and G-I show a partial overlapping of the binding regions of quinidine on hNav1.5 and lidocaine on rNav1.2, respectively.

In the simulations studies performed by Nguyen and colleagues on the homology model of hNav1.5 with lidocaine the most populated site is located just below the SF region. The binding site is formed by the carbonyl groups of residues in DI (T370 and Q371) and DII (C896 and G897) (Nguyen et al., 2019). In this site, lidocaine directly interferes with the binding of sodium in the SF. The existence of such binding site under the SF is also confirmed by the co-crystallization of NavAb with lidocaine and flecainide (El-Din et al., 2018). A feeble density in the same spot was also observed in the cryo-EM structure of rNav1.5 in the presence of flecainide (Jiang et al., 2020) (Figure 5). Finally, a quinidine molecule was observed in that region in the cryo-EM structure of hNav1.5 which was interestingly better resolved than the flecainide molecule (Li et al., 2021a) (Figure 5). This could indicate that flecainide binds less tightly or has a less defined binding site compared to quinidine being in line with a model of blockage by flecainide, where the substance is able to enter the pore during channel opening, then gets trapped within the pore and sterically occludes the pore. Notably, functional studies show a shift of the voltage dependency of fast inactivation for quinidine but not for flecainide (Liu et al., 2002; Pugsley et al., 2005). Furthermore, this quinidine-bound structure also suggests a possible mechanism of action of the molecule: As already observed in the hNav1.7 cryo-EM structure of (Shen et al., 2019), a tyrosine of the intracellular gate (Y1744 in hNav1.7) is stable in two different conformations (Figure 5C). The presence of the quinidine molecule bound just below the SF in hNav1.5 pushes the tyrosine Y1767 in the conformation that is more prone to restricting the intracellular gate (Li et al., 2021a). This is in line with very early experimental work showing that mutations in the SF can directly interfere with LA block of rNav1.4 (Sunami et al., 1997). The recent publication of the cryo-EM structure of rat Nav1.5 bound to propafenone seems to confirm both the experimental and computational studies so far discussed: The drug interacts with rat Nav1.5 *via* the previously-mentioned F1762 and Y1769 (see Table 2 for conversion of numbers between subtypes) *via* its two aromatic rings, and with the residue Q372 in the pore helix in DI, which is part of the most frequently observed lidocaine binding site in Nguyen’s simulations (Nguyen et al., 2019; Jiang et al., 2020).

Nguyen and colleagues also observed the binding of two lidocaine molecules at the same time, suggesting that the pore domain can contain even more than one lidocaine molecule in the uncharged state. The same conclusion was reached from pharmacokinetic studies in which the hill coefficient (which provides a way to quantify the degree of interaction between ligands) for lidocaine binding in Nav1.4 expressed in HEK cells was suggesting either allosteric gating effector properties of LAs or cooperativity (Leuwer et al., 2004). Moreover, the experiments by (Smith, 2006) showed that symmetrical, N-linked lidocaine dimers can be used to block the channel, with the optimal linker being a four-carbon alkylene chain, suggesting the presence of two different bindings sites within the pore.

Two different, LA-charge-dependent states were identified in the work of (Buyan et al., 2018), where the authors use simulations to study the binding of three uncharged (benzocaine, lamotrigine, carbamazepine) and three charged (PF-5215786, PF-6305591, lidocaine) molecules in the pore of prokaryotic Nav from *Magnetococcus marinus* (NavMs). In this case, a first binding site consisting of residues T207 (F1737 in hNav1.7) and F214 (Y1744 in hNav1.7) was identified in helix S6, while the second one is at level of the SF (T176, L177, and E178; NavMs numbering) that attracts the charged ligands (Buyan et al., 2018). Both of the residues belonging to the first binding site were amongst the ones found to reduce tonic block in Ragdale’s experiments on rNav1.2 (Ragsdale et al., 1994; Buyan et al., 2018).

According to these observations, docking studies seem to confirm the presence of different binding modes within the pore, which depend on the protonation state of the LA. An example of this is the study by (Tikhonov and Zhorov, 2017) which proposes the docking pose of charged and electroneutral LA, antiarrhythmics, and anticonvulsants (lidocaine, QX-314, cocaine, quinidine, lamotrigine, carbamazepine, phenytoin, lacosamide, sipatrigine, and bisphenol A) on a homology model of hNav1.4, using NavMs as a template. The channel for the docking was created in two different versions: One with a sodium ion in the Na binding site found at the level of the turn region between the P1 helix and the SF (defined as Na_III_ in (Naylor et al., 2016)), and one where no ion was included. All molecules, independently of their charge, seem to interact with residues in DIV S6 helix (F1586 in hNav1.4; F1737 in hNav1.7) (Tikhonov and Zhorov, 2017). Simulations also seem to indicate that charged or neutral substances binding just below the SF, can block the permeation pathway by an electrostatic or steric mechanism (Tikhonov and Zhorov, 2017). Such findings are in line with simulations conducted on hNav1.5 with lidocaine also binding in the above-mentioned site, where the sodium binding in the SF is decreased (Boiteux et al., 2014; Nguyen et al., 2019). This existence of different binding modes within the pore has further been underlined recently by MD simulations of different antiepileptic drugs and LAs in neutral and charged states. These experiments indicate that charged residues are interacting with the SF and DIV while neutral compounds tend to interact with DII/DIII (Buyan et al., 2021). These data emphasize the complexity of drug/Nav interaction and potentially can explain complex results obtained from mutagenesis studies investigating LA binding sites revealing many different amino acid involved in Nav-LA interaction.

A question that still remains to be answered in detail is the atomistic changes that occur in the binding site regions and access paths (specifically the side-chain interactions with LAs) during different gating states—closed, open and inactivated as predicted by the modulated receptor hypothesis. While available Nav structures become numerous, most of these represent an inactivated state (Yan et al., 2017; Pan et al., 2018; Shen et al., 2019; Jiang et al., 2020).

Even so, the inactivated states have allowed for the elucidation of the crucial role played by the conserved phenylalanine (near the DIII-DIV fenestration) and tyrosine (in DIV-S6 near the activation gate) residues in LA binding. These residues help in stabilizing the LA at the binding site, as discussed in the experimental and computational studies discussed thus far. Presence of a drug also seems to modulate the side-chain rotation of the tyrosine residue, adopting the conformation that is associated with a narrow pore radius (Shen et al., 2019; Li et al., 2021a) The second conformation of this tyrosine residue allows a relatively more open pore in hNav1.7 (Shen et al., 2019). This showcases how the tyrosine residue can control the pore radius and thus could in turn modulate the gating of the channel. A study by Buyan et al., 2021 concurs with this idea that the orientation of this tyrosine side-chain could be a good indicator of the functional state of the channel, showing how the tyrosine adopts a side-chain position pointing towards the central cavity in the inactivated state of hNav1.4, a side-chain position pointing away from the central cavity in purportedly closed NavPaS structures, and towards the intracellular gate in bacterial channels. This could also a play a role in LA binding, since side-chain positions where the tyrosine residue is facing towards the central cavity can take part in interactions with LAs, while this is not possible when it is buried (as in the case of NavPaS and bacterial Navs) (Buyan et al., 2021). The phenylalanine (in DIV S6 near the DIII-DIV fenestration) seems to adopt positions that are not buried, and thus available for interactions in the various structures (in various functional states) (Buyan et al., 2021). This highlights the possible importance of the tyrosine residue in use-dependent block, and the phenylalanine residue in tonic block (Buyan et al., 2021). Navs gate in the time scale of milliseconds (Körner and Lampert, 2020), and hence to obtain gating transitions by MD simulations (with or without LAs) is no small feat. With the techniques of coarse-grained simulations and metadynamics, and also with the deposition of a potentially open-state rNav1.5 channel (Jiang et al., 2021), studying gating transitions at an atomistic level could soon become a possibility in the near future.

## 6 Clinical Perspectives and Future Directions

LAs are broadly used for pain relief during surgery, dental interventions, in obstetrics, and also in the treatment of neuropathic pain (Casale et al., 2017). Especially due to precise injection techniques using ultrasound guided regional anesthesia a patient tailored pain therapy can be established. Many of the currently used LAs bear pharmacokinetic limitations and they don’t meet the clinicians’ and patients’ demand profile closely enough.

As outlined in the chapters above LAs use various routes to access Navs and in some cases need to change their lipophilicity or charge on their way to the binding site. In clinical applications, this may result in the often-observed pharmacokinetic obstacles. The following paragraphs shall give a perspective on the current clinical use, new developments of more subtype selective Nav blockers, and recent developments to improve pharmacokinetic aspects, which may relate to the high lipophilicity of LAs and thus their specific pharmacokinetics.

Development of new LAs is currently tailored for their use in acute local pain but also in pain syndromes, with the aim to produce safer drugs with less sides effects, e.g., as known for opioids. They will address one of our global public health issues (Goldberg and McGee, 2011): While 1 out of 5 adults suffer from pain, the number of patients diagnosed with chronic pain increases up to 10% each year. Acute- and chronic pain is treated by a multimodal approach reaching from educational tools, oral, intravenous as well transdermal pain medication (NSAIDs, opioids, LAs, α2- antagonists, anticonculsants, e.g.,) (Chou et al., 2016). The risks of new persistent opioid medication after surgery as well as chronic opioid medication have to been broadly discussed in the field of the opioid crisis in the United States (Brummett et al., 2017).

### 6.1 Subtype Selective Nav Blockers

Genetic studies have validated the isoforms Nav1.7, Nav1.8 and Nav1.9 as mediators for pain. Especially the identification of congenital insensitivity to pain patients based on loss of function mutations in specific Nav isoforms (Cox et al., 2006) expressed almost exclusively in peripheral sensory neurons sparked the concept of next generation Nav blockers. These could be selective for Nav isoforms expressed in sensory neurons and therefore less prone to CNS and cardiac side effects. Bearing the potential of these substances as LAs and systemic analgesics in mind, major efforts have been made to establish pharmacological accessibility of specific Nav sutypes. The interaction site with the channel had to be distinct from the LA binding site, which is very similar if not identical in most Nav subtypes. One of the first substances meeting the selectivity criterion was the aryl-sulfonamide PF-0589771 introduced by Pfizer, a molecule that interacts with the voltage-sensing domain of Nav1.7 (Alexandrou et al., 2016).

However, although being highly selective for Nav1.7 the development of this compound was discontinued after a failure in a clinical Phase II trial in diabetic neuropathy (McDonnell et al., 2018). In this regard, PF-0589771 shares the fate with of other small molecule inhibitors selective for Nav1.7 (Kingwell, 2019). Despite high target selectivity, pharmacokinetic aspects are not trivial in the targeting of peripheral neuronal Navs, especially when applied systemically. One problem of those early sulfonamide inhibitors is their high fraction of plasma protein association decreasing the effective plasma concentration in clinical trials (Mulcahy et al., 2019). The somata of the sensory neurons are located in the dorsal root ganglia (DRG). Therefore, another aspect is that DRG permeability and different protein binding in DRG tissue compared to plasma may further decrease the effective concentration of the drug (Wu et al., 2017; Luo et al., 2019). Another open question is if systemically applied Nav inhibitors need to penetrate the blood brain barrier as the central projections of the sensory neurons project in the spinal cord and are part of the central nervous system. To date, a variety of Nav blockers considering these aspects are undergoing clinical trials with promising preliminary results (Alsaloum et al., 2020). Remaining challenges are improving the pharmacokinetic properties (plasma-protein-binding, blood-nerve-barrier penetration) which will likely enhance the efficacy of those substances. These pharmacokinetic aspects also arise from the high lipophilicity of these new substances, which they share with the classic LAs. Access to the LA binding site in the Navs requires passage of the watery extracellular space as well as interaction with the lipid phase, as outlined in the chapters above. Thus, devices or procedures that would help distribution or removal of the drugs will improve clinical utility.

### 6.2 Recent Developments to Improve Pharmacokinetic Aspects

Following interventions in which no long lasting postoperative pain is expected, e.g. dental procedures, an “overhang” effect of LAs is undesirable (Prados-Frutos et al., 2015)*.* One option to support ending the effect of LAs is to accelerate the dissociation from the Nav by increased blood flow in the surrounding area by the use of vasodilators such as the α-receptor antagonist phentolamine. This drug has approval in dental interventions for reversal of LA effects when used in combination with vasoconstrictors (Gago-García et al., 2021).

On the other hand, for surgeries in which post interventional pain is expected, often the duration of action of LAs are shorter than the period of postoperative pain. To overcome this shortcoming, continuous application *via* catheters is used for the time of need. The main disadvantages are that the patient is bound to the hospital, and a potential risk of infection. Therefore, several concepts have been developed to achieve longer lasting LA subtypes of which some are already in the clinical pilot stage or clinical use.

One approach is the use of nano-structured drug delivery systems with extended release LAs, e.g., by embedding of LAs into liposomes or polymerosomes. In 2011 liposomal bupivacaine was FDA approved (expanded in 2015) as reviewed in (Malik et al., 2017). In 2020 it was also approved by the EMA. Significantly elevated plasma levels of this compound were measured 96 h after admission when infiltrated in the wound during knee surgery (Bramlett et al., 2012). A direct comparison between epidural application of 0.5% plain bupivacaine with the same amount of a liposomal preparation of the drug showed that the median duration of analgesia was roughly doubled [3.2 h in the control and 6.25 h in the treatment group (Boogaerts et al., 1994)].

Nevertheless, the clinical benefits of this approach are still under discussion. A systematic review investigating the efficacy of liposomal bupivacaine in transverse abdominal plain blocks including seven studies reports a small pain score reduction after 48 h, not after 72 h and no difference in hospital stay (Zhu et al., 2020). As an alternative to liposomes, LAs can also be delivered via polymers. Those macromolecules potentially show more stability on shelf and in co-administration with other LAs as reviewed in (He et al., 2020). Another advantage of polymer delivery is the possibility of controlled delivery, e.g., in a heat dependent manner to deliver LAs especially in inflammatory tissue (Hoare et al., 2012). *In vitro* studies involving chronic compression models in DRG of rats showed steady bupivacaine release from encapsulated in Poly(lactide-coglycolide) (PLGA) nanoparticles for 35 days (Wang et al., 2018).

Beside LAs, the Nav is the target of various neurotoxins. When delivered in small, non-toxic concentrations in combination with LAs, strikingly longer acting time can be achieved. Neosaxitoxin is a Nav blocker produced by dinoflagellates that is thought to bind in the extracellular pore domain of Nav, to a region comprised of the re-entrant pore loop residues (Catterall, 1980). In combination with 10 ml 0.2% bupivacaine and 5 μg/ml epinephrine it showed partial recovery of mechanic detection threshold after 40 and 43 h for cold detection threshold assessed by quantitative sensory testing (Lobo et al., 2015).

An elegant approach to transfer the permanently charged lidocaine derivate QX-314 into the intracellular space uses the open pore of TRPV1 channels (Binshtok et al., 2007). In DRGs, QX-314 had no effect, when given alone, but if it was combined with the TRPV1 activator capsaicin it caused a 2 hours lasting increase in mechanical, thermal and nociceptive thresholds. As TRPV1 is strongly expressed in nociceptors, also a higher target specificity was reached mirrored by the lack of motoric impairment. A further optimized substance BW-031 was shown to be more effective in a UV mouse burn model than QX-314 and was additionally able to decrease cough in an airway inflammation model in guinea pigs (Tochitsky et al., 2021).

In the perioperative environment a fast onset of LAs is highly desirable in order to guarantee immediate anesthesia before the start of an intervention. To fulfill this condition in congruence with sufficiently long lasting anesthesia LA-mixtures of fast-onset-short-duration and slow-onset-long-duration LAs are regularly used (Choi and Gadsden, 2017). The pharmacokinetic advantages of those mixtures are discussed (Choi and Gadsden, 2017). Interestingly, in addition to that, several studies have investigated if modifying the temperature of applied LAs can have an impact on the onset time of anesthesia. Of six studies published between 1987 and 2019 for various blocks and with different substances, four reported a shortened onset time after injection of pre-warmed LAs (Mehta et al., 1987; Heath et al., 1990; Lim et al., 1992; Lee et al., 2012) and only one study reported no effect on onset time (Chilvers, 2019).

## 7 Summary and Outlook

LAs have currently good records in efficacy and safety in medical and dental practice, but they still can have deleterious effects on rare occasions. Knowing the basics of the structures on which LAs bind will help to understand their mechanisms of action. However, the sequence of events that occurs from the administration of LAs to the onset of regional anesthesia-analgesia and/or its toxic effects are complex and depend on very diverse features. These involve the structural and physico-chemical characteristics, the total injected dose, injection site, injection technique, adjuvant drugs, physical condition of the patient, among others. Moreover, ongoing basic research has revealed that LAs can exerts their effects on a multitude of other molecular targets. One example is that lidocaine can affect a wide variety of other proteins, including ion channels, and other targets with pivotal roles in somatosensory neurotransmission and neuroplasticity (Hermanns et al., 2019). As in the case of lidocaine, it is likely that there is not only a Nav-dependent mechanism responsible for its action, but it may be supported by a rather complex synergy of multiple pathways. Furthermore, synergistic effects of LAs in concert with other pharmacological agents used in pain treatment, such as opioids, are likely. Taking again lidocaine as an example, Kir channels, which are insensitive to lidocaine, when tested in combination with opioids, can display a synergistic action (Nockemann et al., 2013).

LAs are very powerful instruments in the clinician’s toolbox. With increasing development of new substances, new drug combinations and delivery systems will provide promising options for continuing improvements in the therapy with LAs. The recent insights from basic research, especially the knowledge gained on the structure-function relations from *in-silico* approaches following experimental testing are cornerstones in this advancement.
